# Effectiveness of Proprioceptive Training on Postural Stability and Chronic Pain in Older Women with Osteoporosis: A Six-Month Prospective Pilot Study

**DOI:** 10.3390/jfmk10030316

**Published:** 2025-08-15

**Authors:** Katya Mollova, Steliyana Valeva, Nazife Bekir, Pavlina Teneva, Kaloyan Varlyakov

**Affiliations:** Department of Health Care, Medical College, Trakia University, Armeiska Street 9, 6000 Stara Zagora, Bulgaria; steliyana.valeva@trakia-uni.bg (S.V.); nazife.sabri@trakia-uni.bg (N.B.); pavlina.teneva@trakia-uni.bg (P.T.); kaloyan.varlyakov@trakia-uni.bg (K.V.)

**Keywords:** osteoporosis, postural stability, balance, coordination, proprioceptive training, fall prevention, chronic pain

## Abstract

**Background**: Osteoporosis is the most prevalent metabolic bone disease, characterized by decreased bone mineral density, which leads to increased bone fragility, back pain, impaired postural stability, and a heightened risk of fractures. Proprioceptive exercises have been identified as an effective approach for reducing the risk of falls and adverse events. **Objective**: Our aim was to conduct a pilot exploratory study evaluating the effectiveness of proprioceptive training in improving coordination and balance, and in reducing chronic thoracolumbar back pain in older women diagnosed with osteoporosis. **Methods**: Quantitative ultrasound bone densitometry was performed on 144 women over the age of 60, followed by the implementation of a proprioceptive training program. The One-Leg Stance balance test and the Visual Analog Scale for pain intensity were administered before and after a six-month training intervention. **Results**: ANOVA revealed significant improvements in balance, with the OLS duration increasing from 2.49 s at baseline to 7.31 s following the intervention. Participants aged over 70 years demonstrated a positive, though comparatively lower increase in stability and balance. Chi-squared (χ^2^) analysis indicated that 83.9% of the variance in OLS performance was attributable to proprioceptive training (Cramer’s V = 0.839, *p* = 0.001). A significant reduction in VAS pain scores was observed, with 48.1% of the variance explained by the moderate effect of proprioceptive training (Cramer’s V = 0.481, *p* = 0.001). **Conclusions**: Proprioceptive training has the potential to improve postural stability, balance, and coordination and stimulate pain intensity in the thoracolumbar region. Despite promising results, the absence of a control group limits our ability to draw definitive causal conclusions.

## 1. Introduction

Bones play a critical role in providing structural support, protection, and facilitating movement in the human body [1]. Peak bone mass is typically achieved during the second decade of a human’s life [2,3]. As we advance in age beyond that point our bone density progressively decreases, resulting in a condition known as osteopenia [4]. When the rate of bone resorption surpasses that of bone formation, bone strength and rigidity become compromised, ultimately leading to osteoporosis [5]. Osteoporosis is a chronic metabolic disease characterized by reduced bone mass and the disruption of the bone microarchitecture, increasing the risk of fractures and functional impairments [1,4]. According to the International Osteoporosis Foundation, osteoporosis represents a significant global public health concern, with a steadily rising incidence associated with fractures and a profound impact on the quality of life in affected individuals [6,7]. Postmenopausal women are particularly vulnerable, exhibiting a significantly higher prevalence of vertebral fractures and chronic back pain [3,8,9]. Hormonal changes after menopause—particularly estrogen deficiency-accelerate bone resorption and reduce bone mineral density. This leads to an increased risk of vertebral compression fractures, which often go undiagnosed but are associated with chronic pain in the thoracic and lumbar regions.

One of the primary consequences of osteoporosis is the deterioration of postural control, balance, and movement coordination, which leads to reduced physical activity, increased risks of falls, and the development of chronic pain [10,11,12]. Numerous morphological and functional changes associated with osteoporotic spinal deformities—such as increased kyphosis and reduced bone density—further compromises stability and motor function [13,14,15,16]. Enhanced thoracic kyphosis shifts the body’s center of gravity anteriorly, thereby altering posture and gait biomechanics. Evidence suggests that impaired proprioceptive function and neuromuscular control in these patients underpin many clinical manifestations, including gait disturbances, instability, and pain [17,18,19].

In recent years, researchers have increasingly focused on the role of physical activity and specific kinesiotherapeutic programs as components of comprehensive osteoporosis management [6,7,20,21,22]. Proprioceptive training, which targets sensory feedback, postural stability, and coordination, has emerged as an effective strategy for reducing the risk of falls and alleviating chronic back pain [23,24]. Studies indicate that such interventions improve neuromuscular control, balance, and overall quality of life in patients with osteoporosis [25,26,27].

Numerous studies have demonstrated that even basic balance exercises—including one-legged standing, controlled weight shifting, and maintaining stability with limited visual input—activate the proprioceptive system by stimulating receptors located in muscles, joints, and tendons [28,29]. Although these exercises are not performed on unstable surfaces, they require constant postural adjustments and sensorimotor control.

Despite the growing body of scientific evidence, data on the optimal parameters and effects of proprioceptive training on balance, coordination, and chronic back pain in this population remain limited. The present study, with its exploratory and pilot nature, aimed to evaluate the effectiveness of a six-month proprioceptive training program on these key aspects in women over the age of 60 with osteoporosis, as well as to explore potential approaches to improving their functional status.

## 2. Materials and Methods

### 2.1. Study Design

This prospective study was conducted over a 24-week (six-month) period with the aim of investigating the relationship between proprioceptive training and balance, coordination, and spinal pain in people with osteoporosis. The study was carried out from November 2024 to April 2025 at the Medical College, Trakia University, Stara Zagora, Bulgaria, as part of the National Scientific Program “Young Scientists and Postdoctoral Fellows-2” of Trakia University-Stara Zagora. This study was conducted in accordance with the Declaration of Helsinki and approved by the Ethics Committee of Trakia University, Stara Zagora, Bulgaria (protocol No. 2/10.10.2024). The target population comprised women over the age of 60 who underwent bone densitometry using a Sonost 3000 ultrasound device, manufactured by OsteoSys Co., Ltd, Seoul, Republic of Korea, year of manufacture 2019 (European representative: Finlink, Helsinki, Finland), to determine the presence or absence of osteoporosis.

All participants underwent the One-Leg Stance (OLS) balance test, and pain intensity was assessed using the Visual Analog Scale (VAS). Each participant was enrolled in a specialized proprioceptive training program, which consisted of targeted proprioceptive exercises performed at home three times per week in 45 min sessions. Participants received instruction on proper exercise execution to maximize efficacy and prevent adverse events. A member of the research team maintained contact with the participants and monitored compliance with the proprioceptive training protocol.

### 2.2. Study Participants

A total of 144 women aged over 60 years were included in the study based on the following inclusion criteria: female patients aged over 60 years, a confirmed diagnosis of osteoporosis, impaired postural control, and functional complaints of chronic pain (lasting more than 3 months) localized in the thoracic and/or lumbar region, without acute inflammatory or neurological symptoms. These complaints lead to reduced mobility and functional difficulties in daily life. The exclusion criteria included: male patients who suffered from osteoporosis, female patients aged under 60 years—in the case of the absence of osteoporosis, fractures having been sustained in the last six months, uncontrolled hypertension or cardiovascular disease, malignancy, myocardial infarction within the last six months, uncontrolled diabetes, the presence of neuromuscular disorders affecting postural control, cognitive impairment impeding understanding of instructions, and other spinal conditions that could compromise the study.

The sample size was determined based on preliminary data and the available literature regarding the effects of balance and proprioceptive programs in similar populations. The goal was to detect a medium to large effect (Cohen’s d ≥ 0.5) with 80% power and a significance level of 0.05. Taking into account the potential dropout rate, a minimum of 100 participants was established.

The participants were provided with information and clarification regarding the purpose of the study. They were informed that their participation was voluntary and that they could withdraw at any time without any consequences. Personal data confidentiality was ensured. All participants gave written informed consent to voluntarily take part in the study.

### 2.3. Research Methods

#### 2.3.1. Balance Assessment

Proprioception refers to the ability to perceive the position and movement of the body in space. The maintenance of coordination and balance is dependent on adequate proprioception [30,31]. To determine the contribution of proprioceptive signals to balance control, various tests are employed. The One-Leg Stance (OLS) test is utilized to assess static balance and postural control. It provides valuable information regarding the risk of falling, the individual’s mobility capabilities, and the efficacy of interventions aimed at improving proprioception and balance. The OLS test requires complex coordination among sensory systems, including visual, vestibular, and proprioceptive input, and involves muscular control, particularly of the ankle, knee, and hip, to maintain stability [30]. Additionally, measurements of the center of pressure (CoP) during OLS offer insights into postural stability and fall risk [32]. OLS is a screening tool for identifying individuals at an increased risk of falls, especially older adults. In our study, we report only the results of the OLS, without accounting for the CoP.

**Figure 1:**
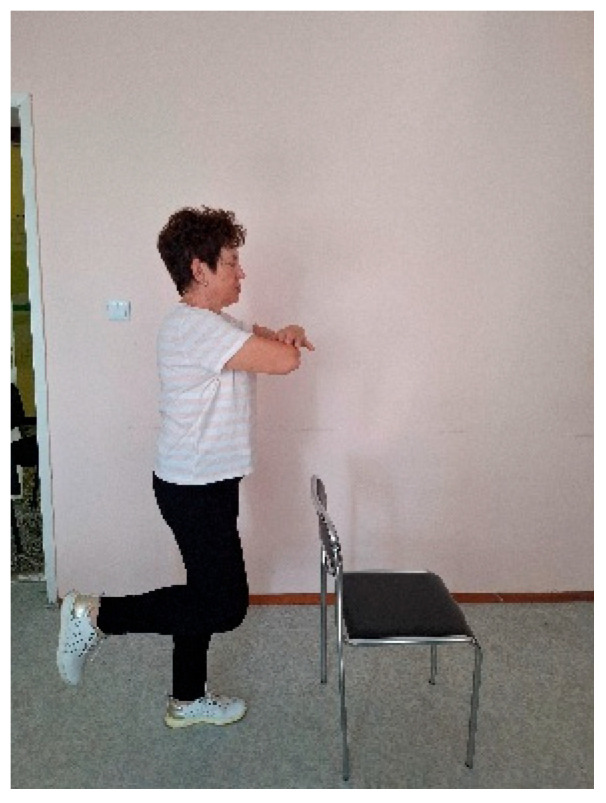
Single-leg balance (OLS) test.

During the OLS test, the participant stands on one leg with arms crossed over the chest, maintaining the position for as long as possible. The test may be performed with eyes open or closed (Figure 1). The duration of the single-leg balance is measured, up to a maximum of 30 s or until the loss of balance.

In this study, the balance assessment was performed in older women with osteoporosis. An inability to maintain the position for at least 10 s is associated with an increased risk of falls [33].

#### 2.3.2. Pain Intensity Assessment

Pain intensity was evaluated using the Visual Analog Scale (VAS) [34]. The scale consists of a 10 cm horizontal line, where “0” represents “no pain” and “10” denotes the “worst imaginable pain” [35]. The results were interpreted according to the following pain severity categories: a score of <3 indicates mild pain, a score of 3–6 indicates mild to moderate pain, and a score of >6 represents moderate to severe pain [36,37]. The pain reported by participants in this study was localized in the thoracic and lumbar regions of the spine. This information was collected through verbal communication with each participant prior to completing the Visual Analog Scale (VAS), with the aim of limiting the subjective assessment specifically to pain in these regions. The assessment was conducted at rest (in a seated position), and participants were asked to indicate the intensity of their pain at the moment of measurement, without direct reference to specific activities such as walking, bending, or standing up.

### 2.4. Proprioceptive Training Program

The participants were enrolled in a six-month specialized proprioceptive training program. The intervention consisted of 40–45 min sessions, conducted three times per week in a home-based setting. The performance of the exercises was periodically monitored and supervised by a rehabilitation specialist through home visits and telephone consultations, to encourage proper technique and motivate participants. The progression of the training load was implemented gradually by increasing the duration of holds, the number of repetitions, or the degree of instability of the support surfaces, taking into account each participant’s individual capabilities and health status.

Of the 150 participants, 144 (96%) completed the full six-month training cycle. Our statistics are based only on participants who completed the full course. Each participant was instructed to complete the program three times a week for 40–45 min per session. The average compliance rate was over 90%, with most participants completing over 70 of the 72 planned sessions.

This study evaluated the effect of a practically applicable training program for older adults, where safety and feasibility are the primary priorities. In this context, a moderately intense physical activity focusing on postural control, coordination, and balance maintenance was chosen.

The exercises were designed to develop the body’s sense of position and movement, as well as to improve neuromuscular coordination.

The overarching goal of the proprioceptive training was to promote the automation of specific motor patterns, thereby enhancing postural control and reducing the risk of falling. The program followed a progressive approach, initially beginning with lower training volumes (6 to 8 repetitions per exercise) and gradually increasing to up to 15 repetitions, in order to avoid overuse and minimize the risk of injury.

To ensure effective proprioceptive stimulation, the program was designed to engage key sensory structures—muscle spindles, Golgi tendon organs, and Pacinian corpuscles—through a variety of exercises involving variable load and instability. Muscle spindles were activated through isometric holds and static balance on unstable surfaces (e.g., air cushions, foam pads, balance boards), which induced constant micro-adaptations and changes in muscle length. Golgi tendon organs were stimulated through low-intensity strength exercises using elastic resistance (Theraband), controlled eccentric contractions, and prolonged isometric efforts that create tension in the musculotendinous junctions. Pacinian corpuscles, sensitive to vibrations and rapid pressure changes, were activated through dynamic exercises with quick position changes and abrupt weight shifts, such as tandem walking, lateral movement, and obstacle negotiation. The combination of different types of movement, the use of unstable supports, and a progressive increase in load created a rich sensorimotor environment aimed at improving postural control and sensory feedback.

All participants received safety instructions prior to beginning the training program, including the following guidelines:

○ All exercises must be performed slowly, with as much control as possible exerted in each movement—focus on the feeling, not the number of repetitions.
○ In the event of pain, dizziness, or discomfort, the exercise should be discontinued immediately.
○ A stable support (e.g., chair, table, cabinet, or wall) may be used if needed for balance assistance, to create a safer environment.

The program included the following key components:

• Warm-up (5 min)
  Light cardio (walking in place or in a straight line with a high knee)—1–2 min;
  Light joint mobility exercises (shoulder, neck and hip rotations)—3 min.
• Stretching exercises (3–5 min) targeting shortened muscle groups, such as cervical extensors, m. pectoralis major, hip flexors and knee flexors;
  Static stretches held for 20–30 s, 2–3 repetitions per muscle group.
• Exercises for coordination, balance, and mobility (25–30 min), including the following:

Static balance training: Patients performed 5–7 exercises on soft and unstable surfaces (gymnastics ball, TheraBand trainers, foam steps, balance cushions and balance)—single-leg stance exercises to improve proprioception; double-leg stance with weight shifting; controlled weight transfer from one leg to the other; performing gentle trunk tilts and rotations while maintaining a static position.

For static exercises, each exercise was performed in 2–3 sets lasting 30–45 s, with 30 s of rest between sets. These were initially performed with visual control and later progressed to eyes-closed conditions upon achieving sufficient postural stability.

Dynamic balance training: This included tandem walking (heel-to-toe along a straight line), obstacle walking, lateral walking, sit-to-stand transitions, squats, and similar functional movements.

Dynamic exercises were performed in 2–3 sets of 10–15 steps or 20–30 s, with 30–45 s of rest between sets.

• Muscle strengthening exercises (12–17 min) were aimed at enhancing muscle groups with observed weakness. These were low-intensity resistance exercises using elastic bands (TheraBand) intended to improve muscular strength. The program targeted the following:
  ○ Lower and upper limb muscle groups—knee extensions; leg curls (knee flexion toward the glutes); arm raises with resistance bands; lateral arm stretches
  ○ Core muscle groups including abdominal, dorsal, and paravertebral muscles—abdominal hollowing (drawing-in maneuver); heel slides; supine marching; glute bridges; modified superman (in prone position, with the alternate lifting of one arm and the opposite leg to enhance back extensor control); bird-dog (from a quadruped position, alternating the extension of opposite arm and leg while maintaining trunk alignment; wall angels; scapular squeezes
  The exercises were performed for 3 sets of 10–15 repetitions per exercise, with 60 s of rest between sets. All exercises were low-impact, performed in a controlled manner, and adapted to individual ability, avoiding spinal flexion or high-torque rotational movements.
• Breathing exercises (static and dynamic) (3–5 min):
  A 5–7 min session included controlled breathing and relaxation.

The rest time between different exercises ranged from 30 to 60 s, adjusted according to individual tolerance and participant condition. In case of fatigue or discomfort, rest periods were extended to ensure safety and maintain proper technique. Some of the exercises can be found in the Supplementary Materials.

### 2.5. Statistical Analysis

Statistical analysis was performed using IBM SPSS Statistics 26.0 (Armonk, NY, USA). To evaluate the effectiveness of proprioceptive training as a method for improving coordination, balance, and motor activity in patients with osteoporosis, the chi-squared (χ^2^) test was used. Statistically significant differences between groups before and six months after the proprioceptive training were determined using one-way ANOVA and univariate ANOVA with the post hoc LSD test. Statistical significance was established at *p* < 0.05. Sample homogeneity was assessed using Levene’s test. The strength of association between categorical variables was determined by calculating Cramer’s V coefficient.

## 3. Results

### 3.1. Participant Characteristics

A total of 144 women were included in the study. The participants were sorted into three age groups: 60–65 years (n = 73, 50.69%), 66–70 years (n = 63, 43.75%), and over 70 years (n = 8, 5.56%) (Table 1).

### 3.2. Analysis of Balance Test (OLS) Results

A one-way analysis of variance (ANOVA) was performed, revealing a statistically significant difference between the mean values of the balance test results prior to and post proprioceptive training (*p* = 0.001). The homogeneity of variances was confirmed by Levene’s test. The mean duration of balance prior to the intervention was 2.49 s (±2.082), with a minimum value of 0 s and a maximum of 9 s. Following six months of proprioceptive training, the mean balance duration increased to 7.31 ± 1.97 s, with a minimum value of 3 s and a maximum of 12 s. These findings indicate a substantial improvement in static balance after six months of proprioceptive training among women with osteoporosis. The significant increase in the duration for which participants were able to maintain the one-leg stance (from 2.49 s to 7.31 s) after six months of proprioceptive training demonstrates the effectiveness of the intervention and indicates improved postural stability and balance (Table 2).

The variations in single-leg stance time (OLS) were categorized by seconds (number (n)) and the percentage (%) of participants in each category, both prior to and post the training program. A statistical analysis of frequency distributions between the two conditions (pre- and post-training) was performed using the chi-squared (χ^2^) test. The effect of the training on the balance retention time was assessed using Cramer’s V coefficient (Cramer’s V = 0.839, *p* = 0.001), indicating a very strong association between the intervention and improvement in static balance, at a significance level of *p* < 0.05. At baseline, 29.2% of participants were unable to maintain balance (OLS = 0 s). After the training program, no participants remained in this category, with the majority progressing to higher time categories. Following the 24-week training period, a significant increase was observed in the number of participants able to sustain balance for longer durations (6–9 s). Balance times of 10–12 s, which were not registered at baseline, were recorded post-intervention. Proprioceptive training resulted in a statistically significant improvement in static balance among the women with osteoporosis in our study. Cramer’s V coefficient confirmed the strong relationship between the intervention and balance test outcomes (Table 3).

### 3.3. Analysis of Pain Intensity Results as Measured by the Visual Analog Scale (VAS)

In the assessment of pain intensity using the Visual Analog Scale (VAS), participants indicated scores ranging from 0 to 6. At baseline, moderate-to-severe pain predominated. However, after six months of proprioceptive training, 54.9% of participants reported no pain or minimal pain (VAS 0–2), with 7.6% reporting the complete absence of pain. Only 0.7% of participants indicated severe pain (VAS = 6). The chi-squared test applied at a significance level of *p* < 0.05 confirmed that the differences in the distribution of pain levels prior to and post the training were statistically significant (*p* = 0.001). The value of Cramer’s V = 0.481 indicated a moderate association between the training program and the reduction in pain intensity. These findings confirm that the observed changes were not due to chance, and the reduction in pain intensity was attributable to the proprioceptive training program (Table 4).

### 3.4. Group Differences

The results of the univariate ANOVA with the post hoc LSD test demonstrated that statistically significant differences (*p* = 0.001) were observed in the mean balance times across all three age groups (60–65, 66–70, and over 70 years) at baseline and six months after the training intervention. For the three age groups, the balance time increased from 2.42 ± 2.02 to 7.16 ± 2.06 s (60–65 years); from 2.73 ± 2.16 to 7.52 ± 1.90 s (66–70 years); and from 1.13 ± 1.55 to 6.88 ± 1.81 s (over 70 years), respectively. Age had a significant impact on balance ability, with the oldest participants (over 70 years) exhibiting lower balance times both prior to and post training. This finding indicates reduced stability and balance within this age group in comparison with the other two. Overall age-related differences remained significant both before and after the intervention. Despite the influence of age on balance capabilities, the proprioceptive training improved overall stability in all groups, as confirmed by the results of the univariate ANOVA (Table 5).

Across the range of balance retention times (0 to 12 s), the distribution of One-Leg Stance (OLS) test results within each age group prior to and post the 6-month proprioceptive training intervention were as follows: At baseline, the highest proportion of participants in the over 70 years group (62.5%) were unable to maintain balance (OLS = 0), compared to 31.5% in the 60–65 years group and 22.2% in the 66–70 years group. After six months of training, there was a marked shift towards higher balance retention times and an increase in the number of participants achieving extended durations (6–12 s), particularly in the 60–65 and 66–70 years groups, and to a lesser extent in those over 70 years of age. A chi-squared analysis was conducted to compare the distributions prior to and post the intervention, and Cramer’s V coefficient was calculated. Prior to the intervention, Cramer’s V = 0.240 (*p* = 0.041) indicating a weak-to-moderate but statistically significant association between age and OLS test results. After six months of proprioceptive training (Cramer’s V = 0.302, *p* = 0.043), the association remained moderate and statistically significant, with greater improvement observed across all age groups, most notably among the younger participants. These results clearly demonstrate that proprioceptive training leads to a substantial improvement in balance retention time in the 60–70 years age groups. The effect is positive, albeit less pronounced, in participants over 70 years of age. This supports the conclusion that the decline in proprioception and balance with age can be improved through targeted exercise. These findings confirm the applicability of proprioceptive exercises for enhancing equilibrium, postural stability, and balance across various age groups (Table 6).

In the analysis of pain intensity (VAS) variations according to age group prior to and post the training program, a high percentage of all groups initially reported moderate to severe pain (VAS ≥ 3), with the highest values observed in the oldest group (over 70 years; VAS 4: 50%, VAS 3: 37.5%). After six months, a reduction in pain was recorded across all age groups. In the 60–65 year group, 7.6% reported no pain (VAS 0), while 20.5% and 28.8% experienced minimal pain (VAS 1 and 2, respectively). A similar trend was observed in the 66–70 and over 70 year groups, with most participants reporting low VAS scores following the intervention.

The results of the chi-squared analysis revealed a statistically significant, albeit weak to moderate, association between age and pain intensity both before the intervention (Cramer’s V = 0.219, *p* = 0.018) and after six months of training (Cramer’s V = 0.136, *p* = 0.046). After six months of training, the association between age and pain intensity diminished but remained statistically significant, likely due to the overall reduction in pain across all groups. The results indicate that proprioceptive training leads to a substantial reduction in pain, with this effect observed in all age groups and with reduced differences between the groups following the intervention. This suggests that the program is effective in pain management and improves the quality of life for women with osteoporosis (Table 7).

## 4. Discussion

This study is a pilot with an exploratory nature, aiming to provide preliminary data on the feasibility and potential effects of proprioceptive training in older women with osteoporosis. The findings may serve as a foundation for future larger-scale and controlled trials. We analyzed the impact of a targeted 24-week proprioceptive training program on balance, coordination, and chronic back pain in women over the age of 60 who suffer from osteoporosis. The results clearly demonstrate significant improvement in static balance, as measured using the One-Leg Stance (OLS) test, as well as a substantial reduction in pain intensity, as assessed using the Visual Analog Scale (VAS). These findings confirm that the aim of the research was fulfilled and extend our current understanding of the role of proprioceptive interventions in osteoporosis management. Furthermore, the present findings are relevant in the selection of rehabilitation programs aimed at fall prevention and the mitigation of adverse outcomes related to fractures in individuals with osteoporosis. Our analyses contribute to the expanding body of literature regarding the necessity of maintaining targeted physical activity for this population.

Adherence to the training regimen likely had a significant impact on the positive outcomes reported in the study. The high compliance rate (over 90%) suggests that participants received an adequate “dose” of the intervention to elicit physiological adaptations such as improved proprioception, neuromuscular control, and reduced pain. Consistent exercise performance likely contributed to thorough motor learning and the consolidation of acquired motor skills. Additionally, regular contact with the rehabilitation specialist may have enhanced participants’ motivation and engagement, further strengthening the intervention’s effect. Thus, the high level of adherence reinforces the validity of the observed results and highlights the program’s potential for real-world application.

A primary limitation of the present study was the absence of a control group, which hindered the establishment of clear causal relationships between the intervention and the outcomes observed. We recommend that future studies employ a randomized controlled design to enable a more precise evaluation of the effects of proprioceptive training on balance and pain in older adults with osteoporosis.

### 4.1. Effects on Balance and Fall Risk

Exercises performed on unstable surfaces elicit a higher stimulation of mechanoreceptors at the level of the tendons, ligaments, and joints, leading to increased proprioceptive feedback to the central nervous system [38]. Dynamic balancing requires the constant adjustment of joint positions and results in enhanced proprioceptive feedback and neuromuscular control. Therefore, proprioceptive exercises increase joint position sense to a greater extent compared to non-specific exercises [39].

The primary finding of this study is the marked increase in balance retention time during the OLS test following proprioceptive training. This improvement can be attributed to the implemented intervention, thereby confirming its positive effect in patients with osteoporosis. The achieved mean improvement of 4.8 s in the One-Leg Stance (OLS) test approaches the commonly accepted threshold of 5–10 s, which is considered clinically meaningful for older adults at increased risk of falls [40]. From this, we can assume that the proposed proprioceptive training program has practical value and leads to effective results.

The One-Leg Stand (OLS) test is a validated and widely used clinical assessment for evaluating balance and fall risk, particularly in older adults. Numerous studies have demonstrated that the ability to maintain balance in this position is a strong predictor of future falls [33,41]. The aim of the proprioceptive training program was not solely to improve performance in this test but to enhance the ability to maintain static balance, which is critical in daily activities such as dressing, standing, and waiting in line. We consider the improvement in this test to be functionally meaningful.

These results are consistent with published meta-analyses and systematic reviews that have also reported significant improvements in balance indicators among individuals with osteoporosis following proprioceptive balance exercises [41,42,43]. Other studies have emphasized the key role of proprioceptive exercises in improving postural stability and reducing fall risks in this population [12,30,44,45,46]. Physical activity incorporating balance and coordination exercises reduces fall risk in older adults by up to 23%. Proprioceptive training leads to improved sensorimotor integration and neuromuscular control by activating receptors such as muscle spindles, Golgi tendon organs, and Pacinian corpuscles. This facilitates postural adjustments, reduces the risk of falls, and enhances balance [47].

Improvements in balance are particularly important in patients with reduced bone mineral density, for whom each fall presents a significant risk of fracture and subsequent complications [6,48]. According to Mikó et al. (2017), sensorimotor balance training significantly enhances postural control, reduces fall incidence, and improves functional capabilities and capacity in women with osteoporosis [49]. This is directly in line with our current findings; the proprioceptive training program led to improvements in postural stability and balance. Such programs also modify the biomechanical characteristics of the spine and gait parameters [22,30], enhancing both static and dynamic stability. This, in turn, increases confidence in movement, reduces fear of falling, encourages physical activity [42,50], and improves quality of life in patients with osteoporosis [51].

### 4.2. Effects on Chronic Pain

VAS data analysis revealed a statistically significant reduction in pain intensity after six months of proprioceptive training, with a greater proportion of participants reporting low pain levels and fewer reporting high pain intensity. Physical activity stimulates the release of endogenous analgesics (e.g., endorphins), increases muscle strength, improves posture, and reduces stress on joints and vertebrae, leading to relief from chronic back pain.

In addition to the statistically significant changes, the results observed exceed the established thresholds for the minimal clinically important difference (MCID), thereby confirming their clinical relevance. In the assessment of pain using the Visual Analog Scale (VAS), the reported mean reduction exceeds 2 points, a value considered in the literature to represent the minimal clinically important difference for patients with chronic pain [52,53].

These results are in agreement with other research indicating that regular physical exercise reduces chronic back pain and improves functional capabilities in individuals with osteoporosis [54,55]. Pain may also be triggered by skeletal deformities, muscle imbalances, and related postural instability [56,57]. Similar findings have been reported by others, who indicate that chronic pain is often a consequence of postural instability [58]. Proprioceptive training improves spinal stability, muscle strength, and postural control, which is particularly important for patients with spinal deformities and back pain [14,22,59]. Pain reduction may also be explained by increased mobility and a reduced fear of falling [60]. In line with our findings, previous authors have emphasized that a regular and targeted training program is essential in achieving meaningful results in managing chronic pain.

### 4.3. Age-Related Differences

The results of subgroup analyses confirm that, while proprioceptive training leads to improvement in all age groups, the positive effect is less pronounced among participants over 70 years of age.

Although the 70+ age group included a relatively small number of participants, which limits the ability to draw reliable conclusions specifically for this age subgroup, its inclusion was designed to provide at least preliminary insights into the effects of the protocol in older individuals and to encourage future studies involving larger and more homogeneous cohorts. Future investigations are expected to include larger and more evenly distributed age groups to allow for more robust and reliable analyses.

The less pronounced effect observed in participants over the age of 70 indicates a progressive decline in neuromuscular function with advancing age. This includes a reduction in both the number and sensitivity of mechanoreceptors—particularly muscle spindles and joint capsule receptors—a slowing of the peripheral nerve conduction, and a loss of fast-twitch motor units.

In adults over the age of 70, polymorbidity is frequently observed (e.g., osteoarthritis, diabetic neuropathy, vestibular disorders), which may limit the capacity for adaptation and neuromotor learning. Age-related sarcopenia reduces both absolute muscle strength and the ability to perform rapid postural corrections in response to instability, potentially constraining the responsiveness to proprioceptive stimulation.

On the one hand, this study confirms an age-related decline in proprioception and balance; on the other, it demonstrates the potential of training interventions, even among older adults [12,61]. This is consistent with previous publications indicating that proprioception and balance ability decline with advancing age and but can nevertheless be improved with appropriate exercise programs [47] or that improved proprioception is a result of regular physical activity [62]. Our analyses revealed a stronger association between age and outcomes for balance and pain at baseline, while after the intervention, this association diminished, likely due to the “equalization” of abilities resulting from the training program.

It is important to emphasize that, despite a comparatively smaller effect observed in the oldest age group relative to younger participants, a meaningful improvement was still recorded, indicating the preserved adaptive potential of the sensorimotor system. In light of these findings, future iterations of the program could benefit from greater individualization and progressive adaptation for participants over the age of 70—for example, by implementing a slower progression in difficulty, extending the stabilization phase, providing additional verbal and visual feedback, and incorporating a longer preparatory period focused on sensorimotor familiarization. Such an approach may enhance the effectiveness of the intervention for this more vulnerable population.

### 4.4. Comparison with Alternative Approaches and Controversies in the Literature

Among the most widely implemented programs for enhancing balance and preventing falls in older adults are the Otago Exercise Program (OEP) and SPEED (Supervised Proprioception Exercise for Elderly with Dizziness). The Otago program comprises a series of progressive lower-limb exercises targeting strength and balance, typically performed under supervision in a home-based setting, and has demonstrated effectiveness in reducing falls among individuals aged 65 and over [63,64]. The SPEED program, in contrast, consists of specialized postural control exercises tailored to older adults with vestibular dysfunction, while also emphasizing improvements in motor function and independence [65].

The present training program shares overarching objectives with these established protocols—namely, improving balance, enhancing functional mobility, and reducing fall risk—but introduces several distinguishing features.

Firstly, the program is intentionally grounded in the principles of proprioceptive stimulation, employing exercises performed on unstable surfaces and incorporating diverse sensorimotor tasks. This approach facilitates the activation of various types of mechanoreceptors and promotes sensory integration, which is critical in maintaining postural stability in older adults with osteoporosis and impaired balance control. Unlike OEP, which emphasizes stable, resistance-based exercises with progressive loading, the current intervention integrates a high degree of sensorimotor variability through the use of air cushions, foam pads, and other dynamic support surfaces.

Secondly, the program is designed to be feasible in a home-based environment without the need for expensive equipment or continuous supervision. This enhances adherence and increases its accessibility to a wider population, including individuals with limited mobility.

Despite the positive trends observed, some studies report that not all types of exercise produce significant effects on balance and pain in osteoporosis, especially when interventions are of low intensity or insufficient frequency [17,66]. Additionally, there is evidence of individual variability in response to training, associated with factors such as motivation, cognitive status, the degree of osteoporosis, and comorbid conditions [10,67]. Certain authors emphasize the need for a multidisciplinary approach that includes both pharmacological (medication) and non-pharmacological therapies (physical activity, dietary interventions, and psychosocial support) [11]. In this context, proprioceptive training should be regarded as a key, but not exclusive, tool in the rehabilitation and prevention of complications in osteoporosis.

### 4.5. Clinical and Practical Implications

The present study supports the incorporation of long-term, structured proprioceptive programs in the rehabilitation of older adults with osteoporosis. Such interventions may reduce the risk of falls and associated fractures, improve independence in daily living, and enhance quality of life [6,47,68]. Furthermore, to achieve pain reduction and balance improvement in patients with chronic pain and impaired postural stability, the early initiation of intervention is crucial. Regular feedback and supervision from the rehabilitation team are essential in the proper execution of the program and the minimization of adverse events [21,69].

### 4.6. Limitations of the Study

Despite its contributions, this study has certain limitations. Among them is the lack of a non-intervention control group. The long-term effects following the completion of the training program, as well as the influence of other factors such as comorbidities and physical activity levels, were not assessed. Another limitation of our study is the absence of measurements related to Center of Pressure (CoP) analysis, which would provide a more precise and quantitative assessment of postural control and balance. The inclusion of these parameters in future research would contribute to an improved understanding of the effects of proprioceptive training and their underlying mechanical mechanisms. Nevertheless, the results clearly highlight the significance of regular proprioceptive exercise in improving balance and coordination and reducing chronic back pain in older women with osteoporosis.

Future research should consider the use of a randomized controlled design and extended follow-up periods, including the evaluation of the long-term sustainability of the observed effects. We also recommend studies investigating combined interventions (proprioception, high-intensity training, etc.) and analyzing their influence on additional outcomes such as fear of falling, quality of life, psycho-emotional status, and functional independence. Future analyses should also incorporate objective measures of dynamic balance, muscle strength, bone density, and biomechanical gait parameters.

## 5. Conclusions

The results obtained suggest that our training protocol with a proprioceptive component, has positive effects on balance, coordination, and chronic back pain in patients with osteoporosis. Despite the limitations of this study, including the absence of a control group and a relatively small and uneven age sample, these preliminary findings support the need for more in-depth and controlled research to confirm the efficacy and safety of such programs.

Programs with a similar profile have the potential to be integrated into comprehensive care for patients with osteoporosis, aligned with current European and global recommendations for prevention and treatment. This work may serve as a foundation for the future development of sustainable health policies and rehabilitation programs aimed at improving quality of life and fall prevention in this growing population.

As a pilot exploratory study, the present work was not designed to provide definitive conclusions but rather to outline directions for future clinical research that can confirm and expand upon these findings.

## Figures and Tables

**Table 1:** Distribution of Study Participants by Age Group.

Age Group	Participants (n)	Percentage of Total (%)
60–65 years	73	50.69%
66–70 years	63	43.75%
>70 years	8	5.56%
Total	144	100%

**Table 2:** Mean Duration of Single-Leg Stance (OLS) at Baseline and Post-Intervention Following Six Months of Proprioceptive Training.

Assessment Period	Mean ± SD (s)	Minimum (s)	Maximum (s)	*p*-Value *
Baseline (n = 144)	2.49 ± 2.082	0	9	0.001
Post-Intervention (n = 144)	7.31 ± 1.973	3	12	

Note: * *p* < 0.05 indicates statistical significance. SD—standard deviation.

**Table 3:** Changes in Single-Leg Stance (OLS) Duration at Baseline and Post-Intervention.

OLS Duration (s)	Baseline (n) %	Post-Intervention (n) %	Total (n) %
0	(42) 29.2	(0) 0.0	(42) 14.6
1	(12) 8.3	(0) 0.0	(12) 4.2
2	(10) 6.9	(0) 0.0	(10) 3.5
3	(38) 26.4	(4) 2.8	(42) 14.6
4	(9) 6.3	(9) 6.3	(18) 6.3
5	(29) 20.1	(14) 9.7	(43) 14.9
6	(1) 0.7	(21) 14.6	(22) 7.6
7	(1) 0.7	(28) 19.4	(29) 10.1
8	(0) 0.0	(24) 16.7	(24) 8.3
9	(2) 1.4	(30) 20.8	(32) 11.1
10	(0) 0.0	(8) 5.6	(8) 2.8
11	(0) 0.0	(2) 1.4	(2) 0.7
12	(0) 0.0	(4) 2.8	(4) 1.4
Total	(144) 100	(144) 100	(288) 100

Note: Cramer’s V = 0.839; *p* = 0.001, indicating a very strong association between the intervention and improvement in static balance.

**Table 4:** Pain Intensity Scores (VAS) at Baseline and Post-Intervention Following Six Months of Proprioceptive Training.

Pain Level (VAS)	Baseline (n) %	Post-Intervention (n) %	Total— (n) %
0	(0) 0.0	(11) 7.6	(11) 3.8
1	(2) 1.4	(26) 18.1	(28) 9.7
2	(19) 13.2	(42) 29.2	(61) 21.2
3	(41) 28.5	(30) 20.8	(71) 24.7
4	(40) 27.8	(25) 17.4	(65) 22.6
5	(27) 18.8	(9) 6.3	(36) 12.5
6	(15) 10.4	(1) 0.7	(16) 5.6
**Total**	**(144) 100**	**(144) 100**	**(288) 100**

Note: Cramer’s V = 0.481; *p* = 0.001, indicating a moderate association between the intervention and the reduction in pain intensity.

**Table 5:** Mean Balance Time (OLS, s) by Age Group at Baseline and Post-Intervention Following Six Months of Proprioceptive Training.

Assessment Period	60–65 Years (n = 73)	66–70 Years (n = 63)	>70 Years (n = 8)	*p*-Value
		Mean ± SD		
Baseline	2.42 ± 2.020 ^a^	2.73 ± 2.164 ^b^	1.13 ± 1.553 ^c^	0.001
Post-Intervention	7.16 ± 2.055 ^a^	7.52 ± 1.900 ^b^	6.88 ± 1.808 ^c^	

Note: Identical letters within each column indicate statistically significant differences between the respective age groups at *p* < 0.05: ^a^ for the 60–65 years group; ^b^ for the 66–70 years group; ^c^ for the >70 years group. SD—standard deviation. Test: post hoc LSD.

**Table 6:** Distribution of OLS Balance Retention Time (s) by Age Group at Baseline and Post-Intervention Following Six Months of Proprioceptive Training.

OLS (s)	Baseline			Post-Intervention		
	60–65 Years (n) %	66–70 Years (n) %	>70 Years (n) %	60–65 Years (n) %	66–70 Years (n) %	>70 Years (n) %
0	(23) 31.5	(14) 22.2	(5) 62.5	(0) 0.0	(0) 0.0	(0) 0.0
1	(5) 6.8	(7) 11.1	(0) 0.0	(0) 0.0	(0) 0.0	(0) 0.0
2	(4) 5.5	(6) 9.5	(0) 0.0	(0) 0.0	(0) 0.0	(0) 0.0
3	(20) 27.4	(15) 23.8	(3) 37.5	(3) 4.1	(1) 1.6	(0) 0.0
4	(3) 4.1	(6) 9.5	(0) 0.0	(5) 6.8	(4) 6.3	(0) 0.0
5	(17) 23.3	(12) 19.0	(0) 0.0	(9) 12.3	(2) 3.2	(3) 37.5
6	(0) 0.0	(1) 1.6	(0) 0.0	(10) 13.7	(11) 17.5	(0) 0.0
7	(1) 1.4	(0) 0.0	(0) 0.0	(13) 17.8	(13) 20.6	(2) 25.0
8	(0) 0.0	(0) 0.0	(0) 0.0	(8) 11.0	(14) 22.2	(2) 25.0
9	(0) 0.0	(2) 3.2	(0) 0.0	(21) 28.8	(9) 14.3	(0) 0.0
10	(0) 0.0	(0) 0.0	(0) 0.0	(1) 1.4	(6) 9.5	(1) 12.5
11	(0) 0.0	(0) 0.0	(0) 0.0	(1) 1.4	(1) 1.6	(0) 0.0
12	(0) 0.0	(0) 0.0	(0) 0.0	(2) 2.7	(2) 3.2	(0) 0.0
Total	(73) 100	(63) 100	(8)/100	(73) 100	(63) 100	(8) 100

Note: Cramer’s V at baseline = 0.240 (*p* = 0.041); Cramer’s V post-intervention = 0.302 (*p* = 0.043).

**Table 7:** Pain Intensity (VAS) by Age Group at Baseline and Post-Intervention Following Six Months of Proprioceptive Training.

VAS	Baseline			Post-Intervention		
	60–65 Years (n) %	66–70 Years (n) %	>70 Years (n) %	60–65 Years (n) %	66–70 Years (n) %	>70 Years (n) %
0	(0) 0.0	(0) 0.0	(0) 0.0	(6) 7.6	(5) 7.9	(0) 0.0
1	(2) 2.7	(0) 0.0	(0) 0.0	(15) 20.5	(9) 14.3	(2) 25.0
2	(9) 12.3	(9) 14.3	(1) 12.5	(21) 28.8	(18) 28.6	(3) 37.5
3	(22) 30.1	(16) 25.4	(3) 37.5	(13) 17.8	(15) 23.8	(2) 25.0
4	(20) 27.4	(16) 25.4	(4) 50.0	(11) 15.1	(13) 20.6	(1) 12.5
5	(9) 12.3	(18) 28.6	(0) 0.0	(6) 8.2	(3) 4.8	(0) 0.0
6	(11) 15.1	(4) 6.3	(0) 0.0	(1) 1.4	(0) 0.0	(0) 0.0
Total	(73)/100	(63) 100	(8) 100	(73) 100	(63) 100	(8) 100

Note: Cramer’s V at baseline = 0.219 (*p* = 0.018); Cramer’s V post-intervention = 0.136 (*p* = 0.046).

## Data Availability

The data presented in this study are available on request from the corresponding author due to privacy and ethical restrictions.

## Supplementary Materials

The following supporting information can be downloaded at https://www.mdpi.com/article/10.3390/jfmk10030316/s1.

## Abbreviations

The following abbreviations are used in this manuscript:

BMD	Bone Mineral Density
VAS	Visual Analog Scale
OLS	One Leg Stance
CoP	Centre of Pressure
