# Supplementary material for: Effectiveness of Proprioceptive Training on Postural Stability and Chronic Pain in Older Women with Osteoporosis: A Six-Month Prospective Pilot Study
Source: J Funct Morphol Kinesiol. 2025 Aug 15;10(3):316. doi: 10.3390/jfmk10030316 (PMC12371942; doi:10.3390/jfmk10030316)

Proprioceptive Training Program for Women with Osteoporosis

1. Stretching Exercises for Shortened Muscle Groups

Instruction: *Elongate limbs along their longitudinal axis and hold in the stretched position for 2–3 seconds; each stretch is performed for 5–8 repetitions.*

1. 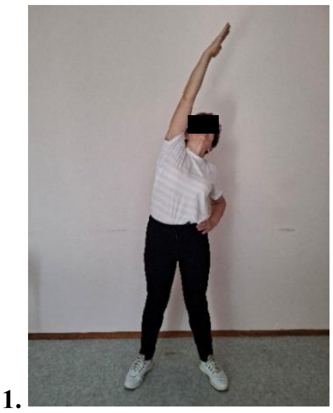 **2.** 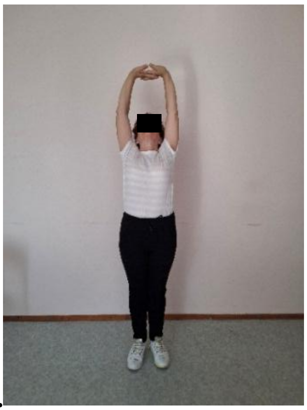 **3.** 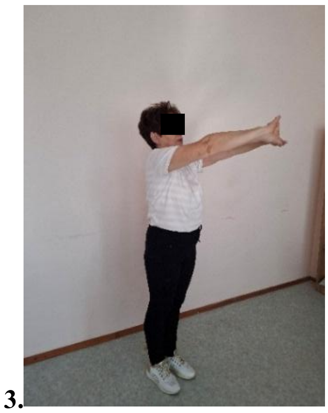 **4.** 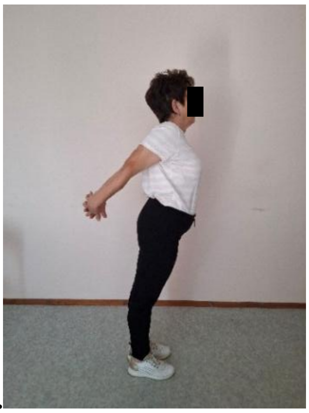

2. Exercises to Improve Proprioception, Coordination, Balance, and Mobility

Static Proprioceptive Exercises.

Instruction: *Perform the below exercises initially with eyes opened hold for up to ten seconds, depending on your abilities. Once the technique is performed in a comfortable manner, repeat the exercise with eyes closed.*

5. 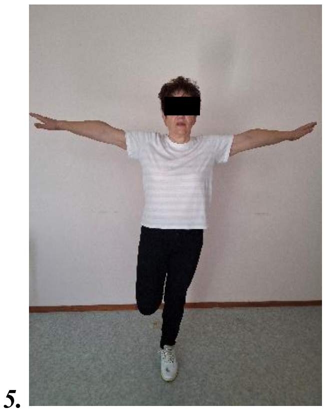 **6.** 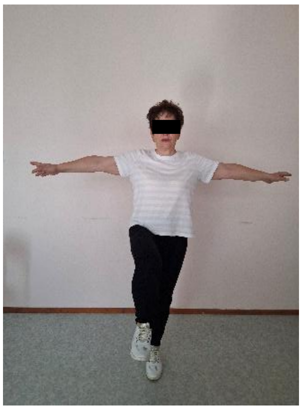 **7.** 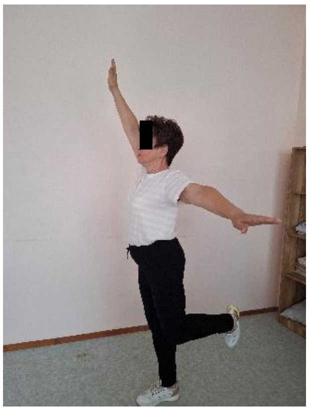

Dynamic exercises

Exercise 8: Sit-to-stand transitions from a chair – 5 repetitions.

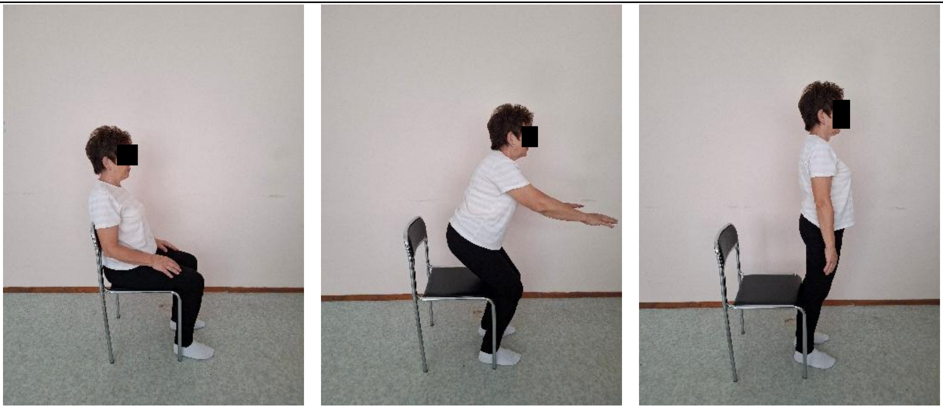
8.

Exercise 9: Tandem walking in a straight line – approximately 10 meters.
Exercise 10: Straight-line walking with obstacles – approximately 10 meters.
Exercise 11: Lateral walking with obstacles – approximately 10 meters.

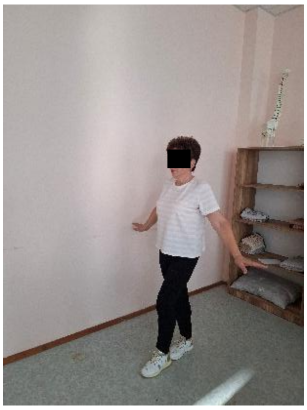
9.

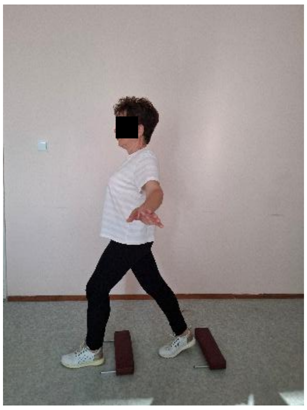
10.

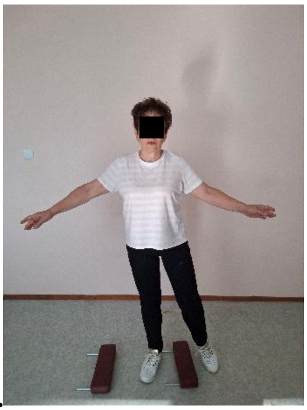
11.

Exercise 12: *Supported half-squat – 6 to 10 repetitions. This exercise aims to strengthen the lower limb muscles while maintaining joint stability.*
Exercise 13: *Contralateral arm and leg extension in multiple directions – 6 to 10 repetitions. Designed to improve core stability, coordination, and functional strength.*
Exercise 14: *Step-up and step-down repetitions on a single step – 5 repetitions.*

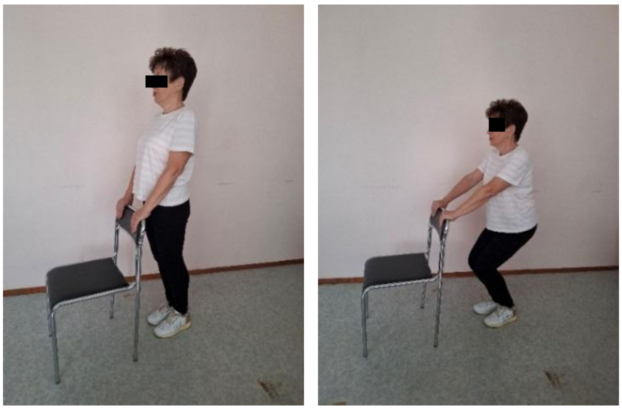
12.

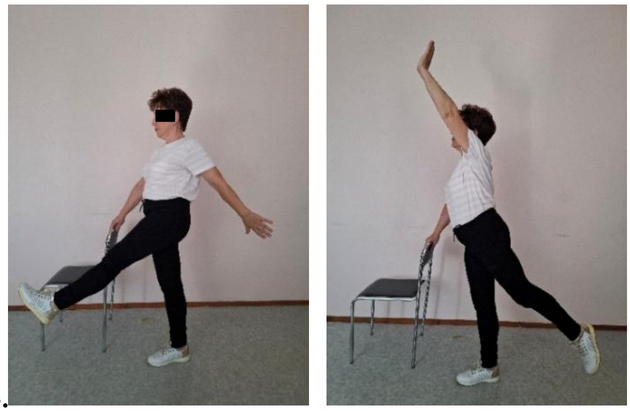
13.

14. 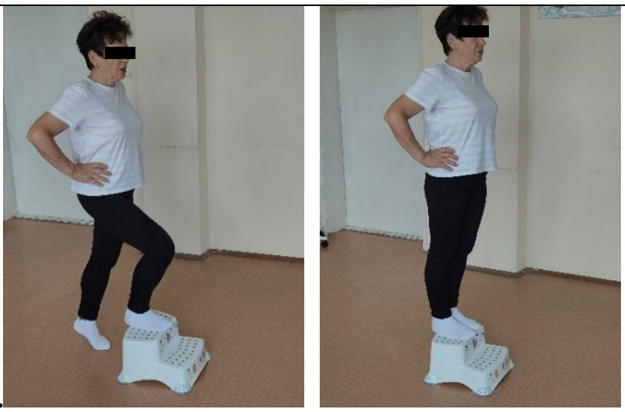

3.Focused exercises on enhancing lower limb strength, balance, and motor control.
These are low-intensity resistance exercises, performed with or without external resistance (e.g. elastic bands), aimed at strengthening weak muscle groups.
Instructions: *Perform the exercises slowly, within the available range of motion. During the initial two to three months, 6–10 repetitions are recommended. In the following months, increase the number of repetitions gradually up to 15.*

- ***Exercises for Strengthening Upper Limb Muscles***

21. 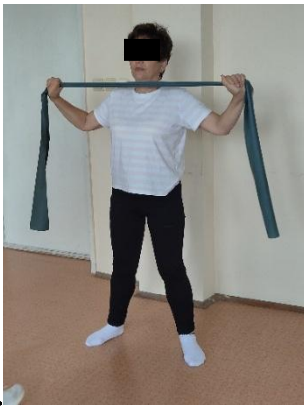
22. 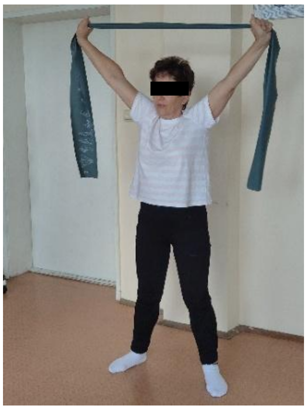
23. 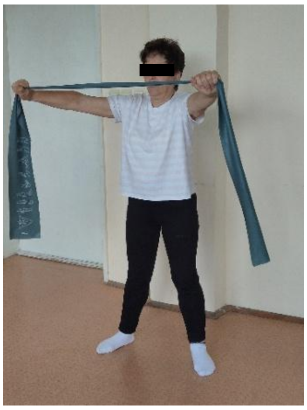
24. 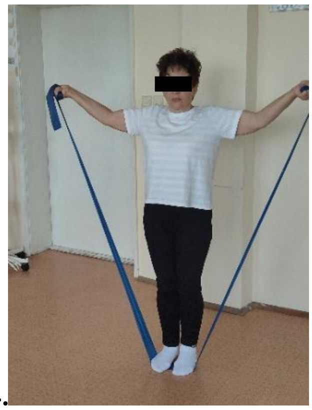
25. 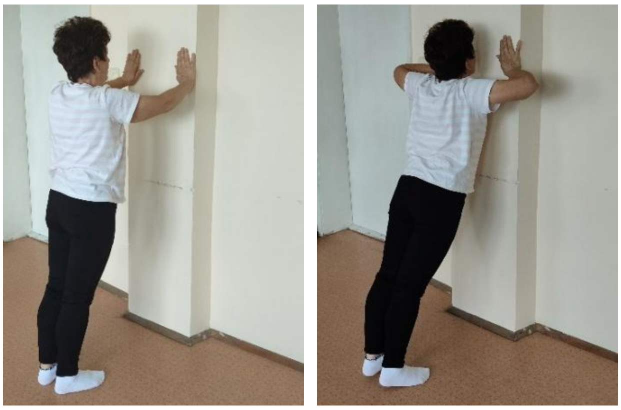

- ***Exercises for Strengthening Lower Limb Muscles***

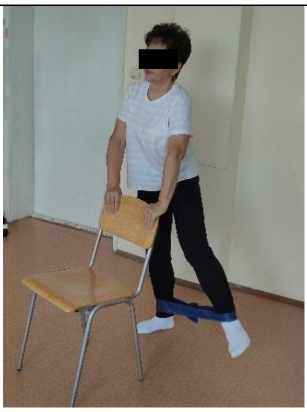

26.

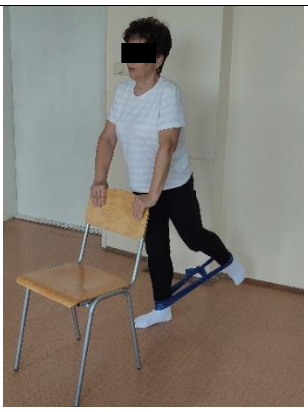

27.

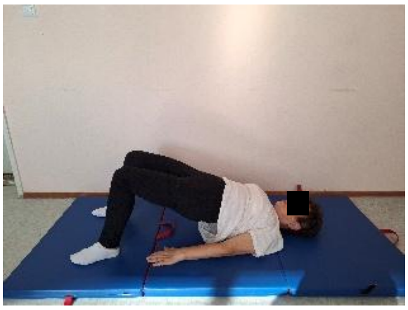

28.

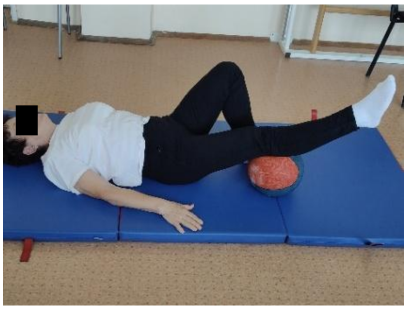

29.

- ***Exercises for Strengthening Abdominal Muscles***

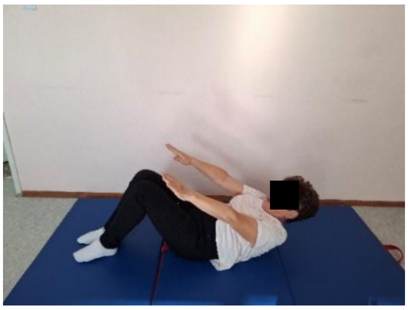

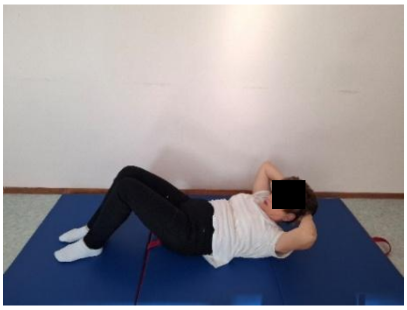

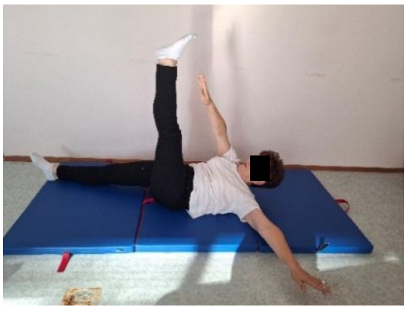

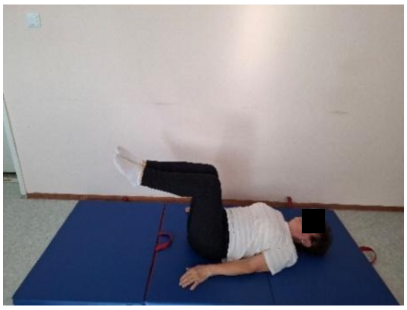

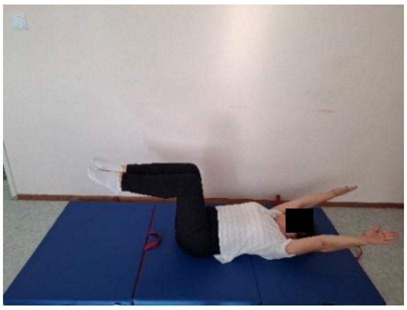

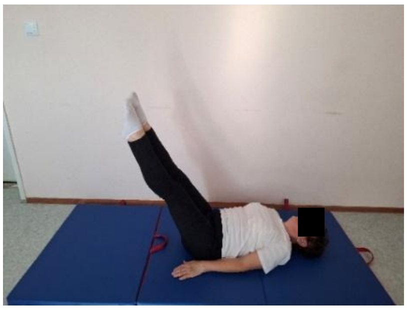

- ***Exercises for Strengthening the Dorsal and Paravertebral Muscles***

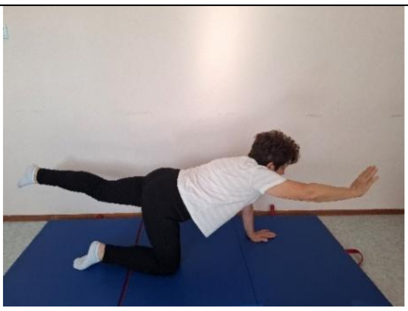
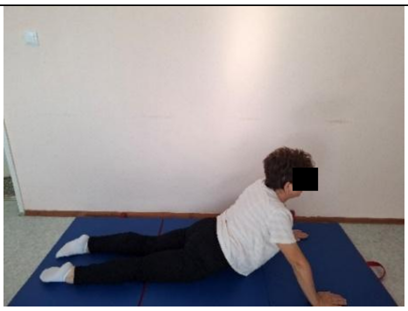
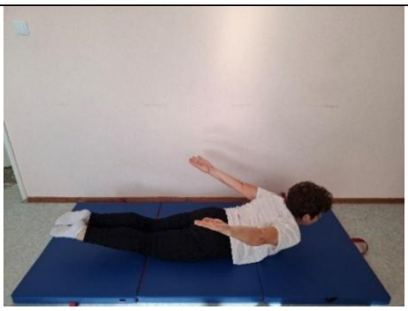
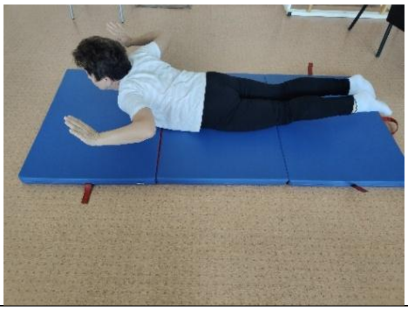
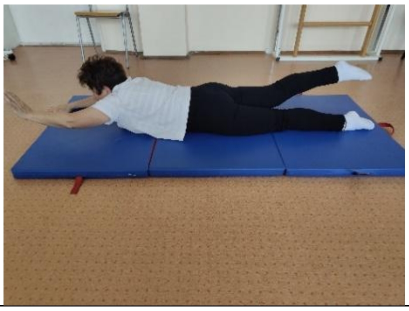

Relaxation Exercises

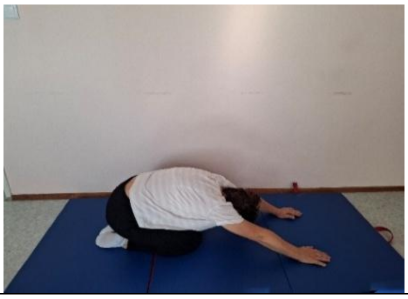

Supplement: Supplementary file 1 [file jfmk-10-00316-s001.zip › jfmk-3779987-supplementary.pdf]
